# Comprehensive identification of immune-associated biomarkers based on network and mRNA expression patterns in membranous glomerulonephritis

**DOI:** 10.1186/s12967-018-1586-4

**Published:** 2018-07-24

**Authors:** Chengwei Zhang, Lei Leng, Xiaoming Zhang, Yao Zhao, Zhaozheng Li

**Affiliations:** 1 Department of Nephrology, The Second Affiliated Hospital of Harbin Medical University, Harbin, 150006 Heilongjiang People’s Republic of China; 2 The Second Hospital of Harbin, Heilongjiang, 150006 People’s Republic of China; 3 Xiangan Hospital, The First Affiliated Hospital of Xiamen University, Xiamen, 710000 Shanxi People’s Republic of China

**Keywords:** Membranous glomerulonephritis, Immune, Network modules, Topology feature, Biomarker

## Abstract

**Background:**

Membranous glomerulonephritis (MGN) is the most common cause of nephrotic syndrome in adult patients. Despite extensive evidences suggested that many immune-related genes could serve as effective biomarkers in MGN, the potential has not been sufficiently understood because of most previous studies have concentrated on individual gene and not the entire interaction network.

**Methods:**

Here, we integrated multiple levels of data containing immune-related genes, MGN-related genes, protein–protein interaction (PPI) networks and gene expression profiling data to construct an immune or MGN-directed neighbor network (IOMDN network) and an MGN-related genes-directed network (MGND network).

**Results:**

Our analysis suggested that immune-related genes in the PPI network have special topological characteristics and expression pattern related to MGN. We also identified five network modules which showed tighter network structure and stronger correlation of expression. In addition, functional and drug target analyses of genes in modules indicated that the potential mechanism for MGN.

**Conclusions:**

Collectively, these results indicated that the strong associations between immune and MGN and showed the potential of immune-related genes as novel diagnostic and therapeutic targets for MGN.

**Electronic supplementary material:**

The online version of this article (10.1186/s12967-018-1586-4) contains supplementary material, which is available to authorized users.

## Background

Membranous glomerulonephritis (MGN) is one of immune-mediated and more common forms of nephrotic syndrome in the adult population [1, 2]. MGN shows a special type of immune complex glomerulonephritis and had the symptoms of glomerular subepithelial IgG-containing immune complex deposits and usually heavy proteinuria [3, 4]. Previous study had confirmed that primary MGN is an autoimmune disease and M-type phospholipase A2 receptor (PLA2R) is a major specific antibody in this disease [5]. For early-childhood membranous nephropathy, other study also show cationic bovine serum albumin as the target antigen of antibodies deposited in experimental models [6]. However, the etiology and mechanism of MGN are unknown in most patients and also designated as idiopathic membranous glomerulonephritis in the past. Therefore, it’s essential to identify novel signatures or biomarkers that can enhance clinical behaviors in the treatment of MGN.

A large number of previous studies report that the associations between immune and MGN. MGN is thought to occur due to immunoglobulin and related with other autoimmune conditions [7–9]. MGN usually develop with Th2-Type Immune deviations in MRL/lpr Mice Deficient for IL-27 Receptor [10]. Although the MGN is closely related with immune, the mechanisms for formation and deposition of auto-antibodies in human MGN remain unknown. Especially, most of our current knowledge on mechanisms for MGN is derived from studies in experimental models [9].

Genetic factors are also likely to contribute to the process of MGN. Polymorphisms of genes could become as attractive candidates for elucidating the clinical variability of MGN [11]. For example, the different genotypes of NPHS1 are associated with susceptibility of MGN and the remission of proteinuria during disease progression after the therapy [12]. The polymorphisms of STAT4 are associated with susceptibility to primary membranous glomerulonephritis and renal failure [13]. Cerebral sinovenous thrombosis associated with factor V Leiden and methylenetetrahydrofolate reductase A1298C mutation are explored in adult membranous glomerulonephritis [14]. In addition, there are also some genes were differential expressed in MGN or membranous nephropathy [15, 16]. These findings demonstrate that identify key genes could help study the mechanism and therapy of MGN. However, these studies almost focus on one or a few genes analyzed in either cell lines or limited patient samples. Previous studies had reported the advance of integrating the gene expression and protein–protein interaction data to identify key genes in diseases [17, 18].

In this study, we constructed an immune or MGN-directed neighbor network (IOMDN network) and an MGN-related genes-directed network (MGND network) to address the role of the immune network in MGN. We identified immune-related genes in the PPI network with hub topological features connected to MGN. We also analyzed the topological and expression patterns of IOMDN network. We identified five modules from the MGND network and had special expression patterns. These modules included several key immune-related and MGN-related genes, demonstrating the functional significance of the immune-related genes to MGN. A functional and drug target analysis revealed that the genes in these modules were co-associated with immune system activation and other processes in MGN. Our findings highlighted the novel role of the immune-directed network in MGN. These comprehensive analyses can serve as important resources for future experimental dissection of biomarkers in MGN.

## Methods

### Human immune-related gene datasets

We download all the genes related with immune from AmiGO 2 version: 2.4.26 in Homo sapiens species, constituting 3068 immune-related genes from 651 records [19].

### Membranous glomerulonephritis-related gene datasets

The MGN-related genes were downloaded from DisGeNET, which is a discovery platform containing one of the largest publicly available collections of genes and variants associated to human diseases. The database collected 561,119 gene-disease associations, between 17,074 genes and 20,370 diseases, disorders, traits, and clinical or abnormal human phenotypes [20]. At last, we got 90 MGN-related genes including the records labeled as membranous glomerulonephritis and idiopathic membranous glomerulonephritis.

### Human protein–protein interaction data

The protein–protein interaction (PPI) data for constructing the human protein interaction network were download from the HPRD (Human Protein Reference Database) database [21]. More than 42,000 manually curated interactions between 9826 human genes were contained in the database.

### The construction of network and analysis of topological characteristics

First, a human PPI network associated with immune and MGN was constructed based on the HPRD data. Then, a sub-network of the human PPI network named the immune or MGN-directed neighbor network (IOMDN network), which including immune or MGN-related genes and their direct interacting genes in the network (referred to as immune neighbor genes) using all the immune-related genes and MGN-related genes from AmiGO and DisGeNET. Finally, we extracted all MGN-related genes to construct an MGN-related genes-directed network (MGND network) for subsequent analysis. We used cytoscape software to construct network and analyze the topological properties of nodes in both IOMDN and MGND network.

### Identifying modules from MGND network

We identified all the network modules using MCODE follow the default parameters based on the MGND network constructed by the MGN-related genes (http://apps.cytoscape.org/apps/mcode). MCODE is a tool which clusters a given network based on topology to find densely connected regions. We identified 20 modules and extracted the five modules with top numbers of nodes.

### Integration of MGN gene expression by clinical datasets

We obtained the expression data of MGN from GEO (Gene expression Omnibus). The MGN expression profile data included 21 diseases samples and 18 control samples (GSE99340) [22]. We calculated the Pearson correlation coefficients (PCCs) between two nodes either in MGND network or modules used by disease samples expression data. We also used unpaired t-test to get the p values of differential expression genes between disease and control samples for all the nodes in the modules.

### Gene set enrichment analysis

Gene ontology (GO) and KEGG pathway enrichment analyses were performed by the DAVID functional annotation web server using default parameters [23]. We obtained enriched GO terms and pathways (p < 0.01).

### Drug enrichment analysis

With the Enrichr tool online web server using default parameters, drug enrichment was performed for all the genes in five modules. We obtained enriched drugs (p < 0.01) [24].

## Results

### Immune-associated genes play a crucial role in membranous glomerulonephritis

**Fig. 1:**
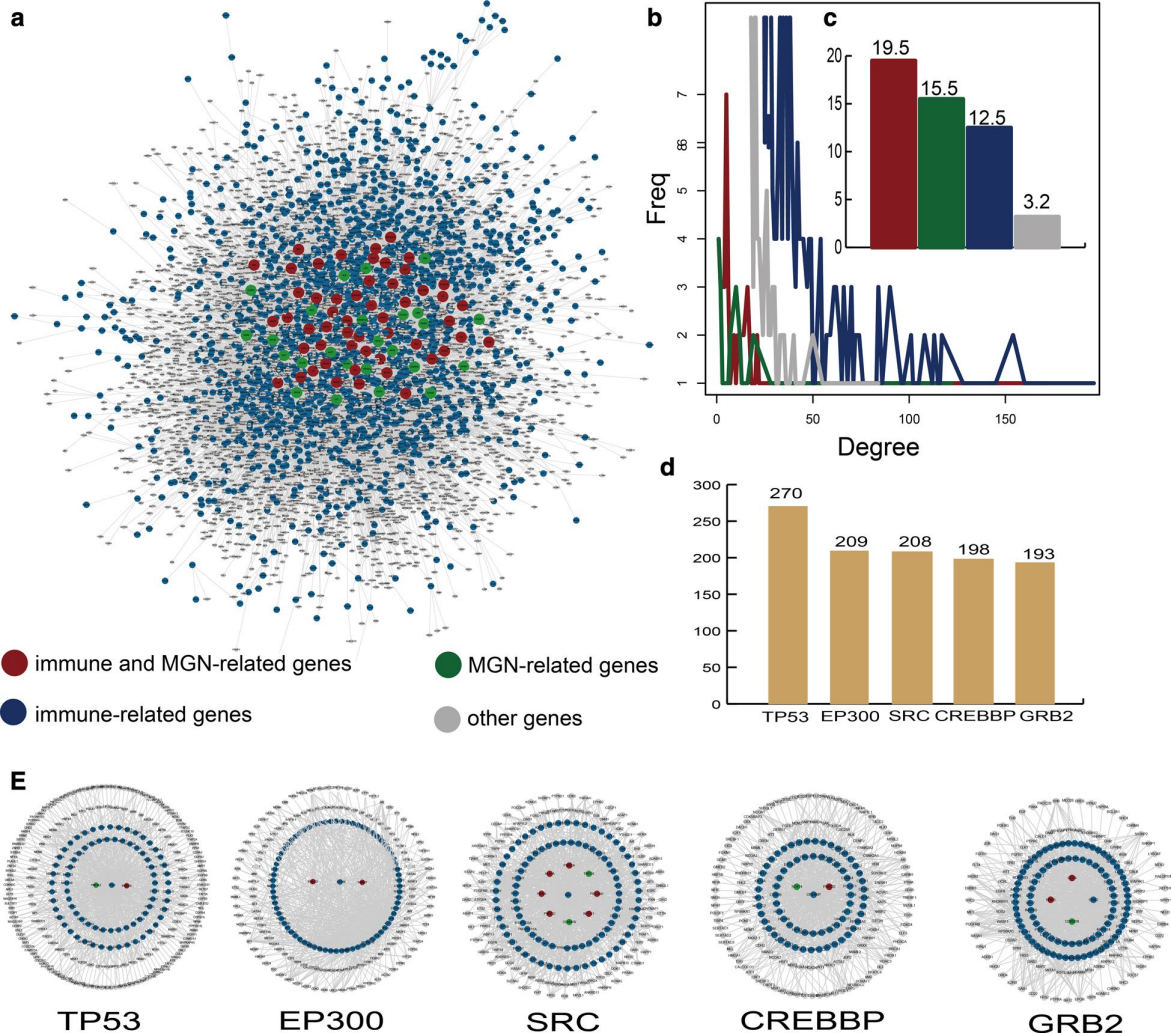
The properties of immune or MGN-directed neighbor network (IOMDN network). **a** The global IOMDN network. **b** The degree distribution of immune and MGN-related genes, MGN-related genes, immune-related genes and other genes, respectively. **c** The barplot shows the average degree of diverse kinds of genes. **d** The top five genes rank by gene degree including TP53, EP300, SRC, CREBBP and GRB2. The barplot shows the degree of the five genes. **e** The sub network of top five genes and their direct neighbors

A sub-network of the human PPI network named the immune or MGN-directed neighbor network (IOMDN network) is constructed and the IOMDN network contains 6251 nodes and 19,983 edges (Fig. 1a). We find 56 immune-related genes that are themselves MGN-related genes such as MMP, CTLA4, CD19 and so on. We also discover 34 only MGN-related genes and 3011 only immune-related genes (Additional file 1: Table S1). We analyze the degree distribution of immune and MGN-related genes, immune-related genes, MGN-related genes and other genes, respectively. All the degree of these genes show the scale-free distribution (Fig. 1b). Especially, we further find a higher degree of genes (mean = 19.5) that were both related with immune and MGN in the IOMDN network and indicating complex links between the immune-related genes and MGN-related genes (Fig. 1c). The top five genes including TP53, EP300, SRC, CREBBP and GRB2 which are all immune-related genes have a direct connection and highest degree in the network, indicating that immune-related genes play hub roles in the IOMDN network (Fig. 1d). Notably, these five top immune-related genes are all interacted with some MGN-related genes (Fig. 1e). For example, immune-related gene SRC directly connects with MGN-related gene including TPRC6, CDKN1B, PRKCA and so on. All above results indicate that immune-related genes play crucial roles in membranous glomerulonephritis.

### Immune-associated genes directly interact with membranous glomerulonephritis-related genes

**Fig. 2:**
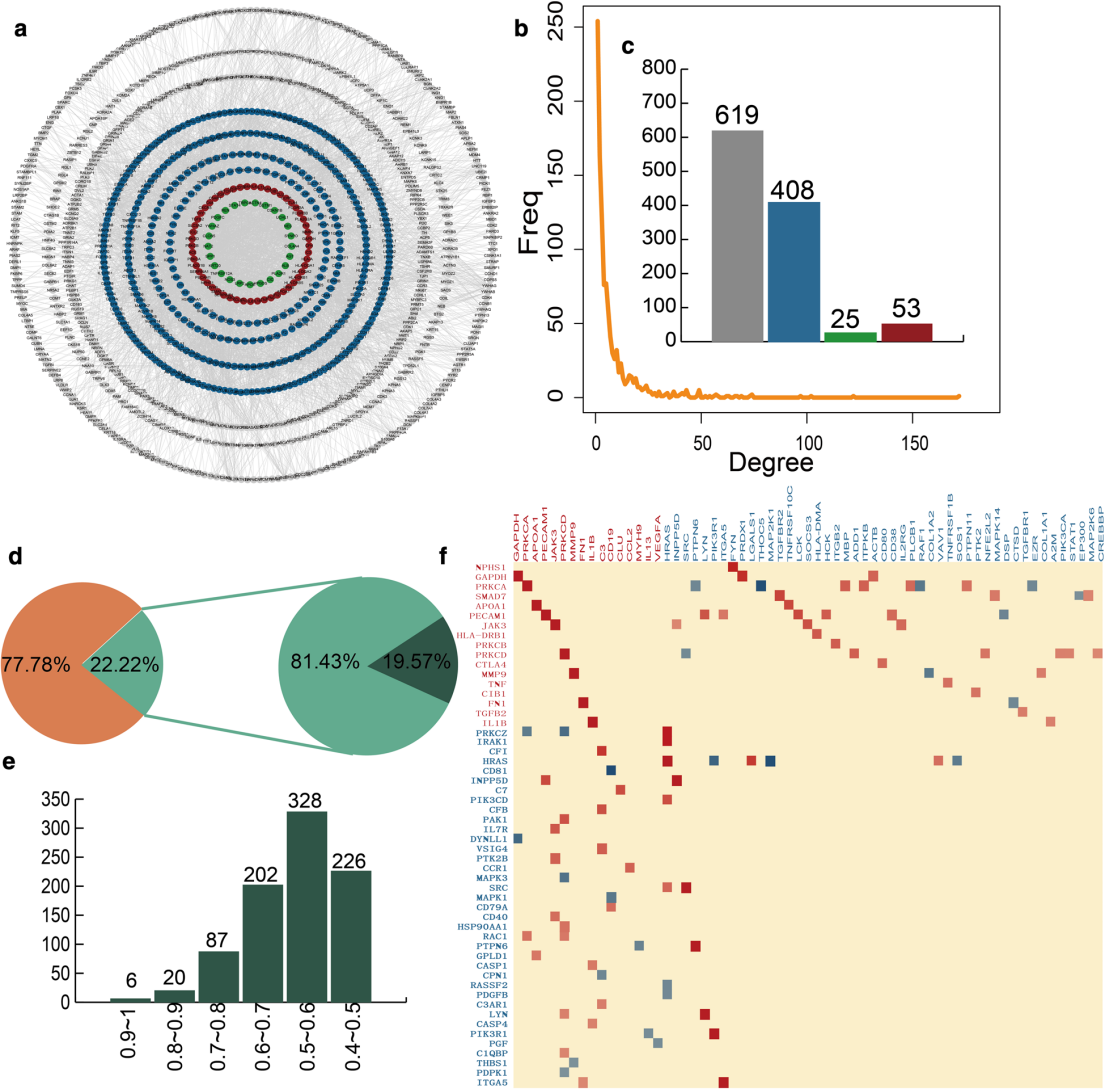
The characteristics of MGN-related genes-directed network (MGND network). **a** The global MGND network. **b** The degree distribution of all nodes in MGND network. **c** The number of immune and MGN-related genes, MGN-related genes, immune-related genes and other genes, respectively. **d** The first pie chart shows the percent of interaction in MGND and IOMDN network. The second pie chart shows the percent of significant correlated correlation interactions. **e** The numbers of interactions across diverse levels of PCC values. **f** The heatmap of PCC values between immune and MGN-related genes and immune-related genes

We construct a network named MGN-related genes-directed network (MGND network) to further explore the relationship between immune-related and MGN-related genes (Fig. 2a). This sub network is extracted from the IOMDN network by selecting the MGN-related genes and their directly connected genes. We find the MGND network also show scale-free distribution (Fig. 2b). There are 53 immune and MGN-related genes, 25 only MGN-related genes, 408 only immune-related genes and 619 other genes in MGND network (Fig. 2c). Compared to the topological features of nodes in IOMDN, the nodes in MGND have higher network density (avg. 0.007 vs 0.001), degree (avg. 8.009 vs 6.389) and clustering coefficient (0.154 vs 0.097). This result indicates that the MGND extracted from IOMDN is closer in structure and played a crucial role in the biogenesis of MGN.

Next, we integrate gene expression data for the MGND network to further study the relationships between MGN-related and immune-related genes on expression level. The interaction relationships in MGND hold 22.22% of interactions in IOMDN. 19.57% interactions are significantly co-expression in all the interactions of MGND network (Fig. 2d). We also find 643 (73.99%) significantly co-expressed interactions in MGND and show strong correlation (Pearson correlation coefficients > 0.5) (Fig. 2e). The strong correlations between the nodes in MGND network indicate that the association between immune-related genes and MGN-related genes not only show on topological structure but also show on expression pattern. We further find immune and MGN-related genes are significantly correlated with a large number of immune-related genes (Fig. 2f). For instance, immune and MGN-related gene NPHS1 is strong correlated with immune-related gene FYN (PCC = 0.88). In addition, these significant correlations contain both positive and negative correlation. The MGN-related gene PRKCA is positive correlated with MBP and ITPKB (PCC = 0.61, 0.6), however, negative correlated with PTPN6 and THOCA5 (PCC = − 0.48, − 0.74). The results show that the complex properties between the communication of MGN-related genes and immune-related genes.

### Immune genes associated with membranous glomerulonephritis genes are related on expression level based on modules

**Fig. 3:**
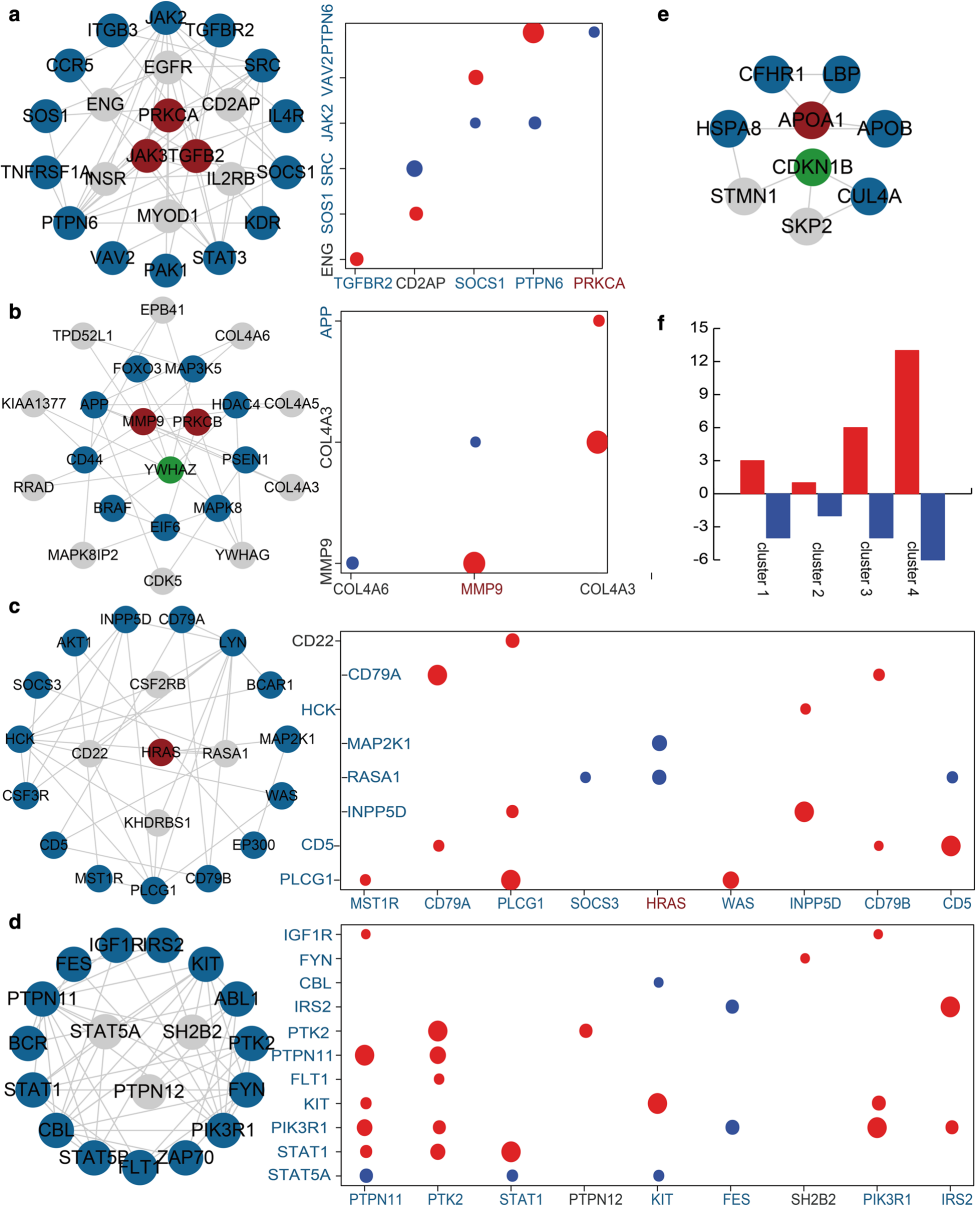
The five modules identified from MGND network. **a**–**e** The sub network of five modules and the color are showed as Fig. 1. The co-expression pattern of each module, the larger circle represents stronger correlation and red represents positive correlation, blue represents negative correlation. **f** The number of positive correlations and negative correlations for each module

We perform a module analysis for the MGND network to reveal the communication between immune-related genes and MGN-related genes. We extract five modules include top number of nodes and also analyze their expression patterns (Fig. 3). The first module contains three immune and MGN-related genes, 14 immune-related genes and six other genes. Integrating expression data, we find MGN-related gene PRKCA has strong negative correlation with immune-related gene PTPN6 (PCC = − 0.48, p = 0.027) (Fig. 3a). The second module included two immune and MGN-related genes, one MGN-related gene, eight immune-related genes and 10 other genes (Fig. 3b). The genes MMP9 and APP were also related with other genes on expression level. There were one immune and MGN-related gene, 15 immune-related genes and four other genes in the third module. The most of immune-related and MGN-related genes in third module had strong positive and negative correlations (Fig. 3c). For example, immune and MGN-related gene HRAS were negative correlated with two immune-genes. The fourth and fifth modules also contained strong interaction relationships (Fig. 3d, e). We also discovered the direction of correlation was complex in four modules (Fig. 3f).

### Identification of differential expressed immune-related modules and genes associated with membranous glomerulonephritis

**Fig. 4:**
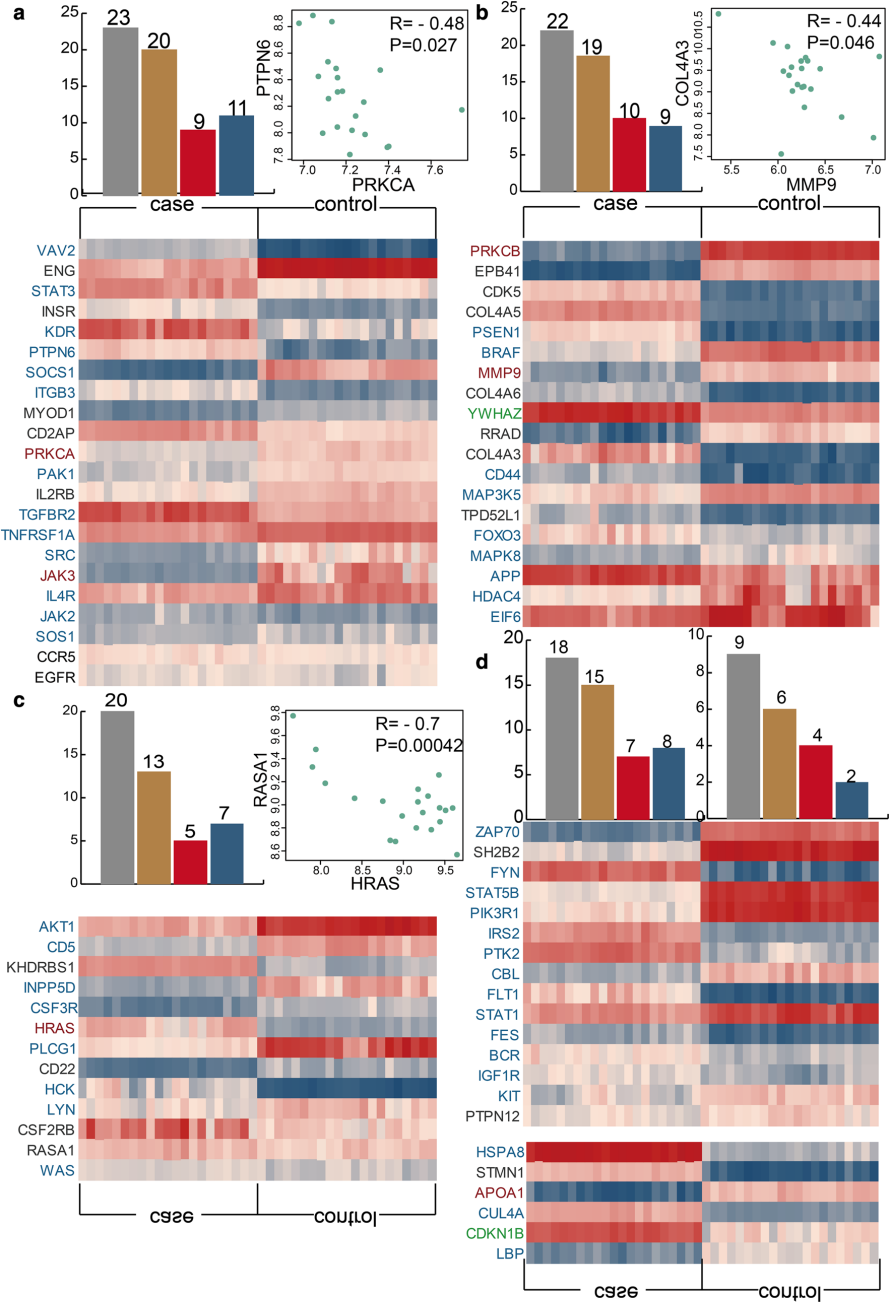
The differential expression patterns of five modules. **a**–**d** The bar plots show the number of all genes, differential genes, up-regulated genes and down-regulated genes for each module. The point plots show the expression pattern between two significant gene interactions. The heat maps show the differential expression between MGN patients and normal samples for all genes in five modules

After exploring the interactions between immune-related genes and MGN-related genes in five modules, we also consider if there are differential expressed genes for MGN patients and control samples. By integrating expression data, we discover diverse number of differential genes in all the modules. We find 20 differential genes including nine up-regulated and 11 down-regulated genes in all the 23 genes for the first module (Fig. 4a). Notably, the differential expressed MGN-related gene PRKCA is negative correlated with immune-related gene PTPN6. For the second module, 19 genes of all the 22 genes are differential expressed contained ten up-regulated and nine down-regulated genes (Fig. 4b). There are 12 differential genes including five up-regulated and seven down-regulated genes. We also discover that immune and MGN-related gene HRAS is strongly negative correlated with immune-related gene RASA1 (PCC = − 0.7, p = 0.00042) (Fig. 4c). The fourth and fifth module also show the similar phenomenon (Fig. 4d). The differential expression details of all genes in five modules are showed in Additional file 2: Table S2. Our results show that some immune-related and MGN-related genes are differential expressed between MGN patients and control samples and these differential expressed immune-related genes and MGN-related genes are co-expressed in MGN patients. It indicates that the immune-related genes are associated with MGN-related genes on expression level.

### The functional and drug target analysis shows the roles of immune-related modules in membranous glomerulonephritis

**Fig. 5:**
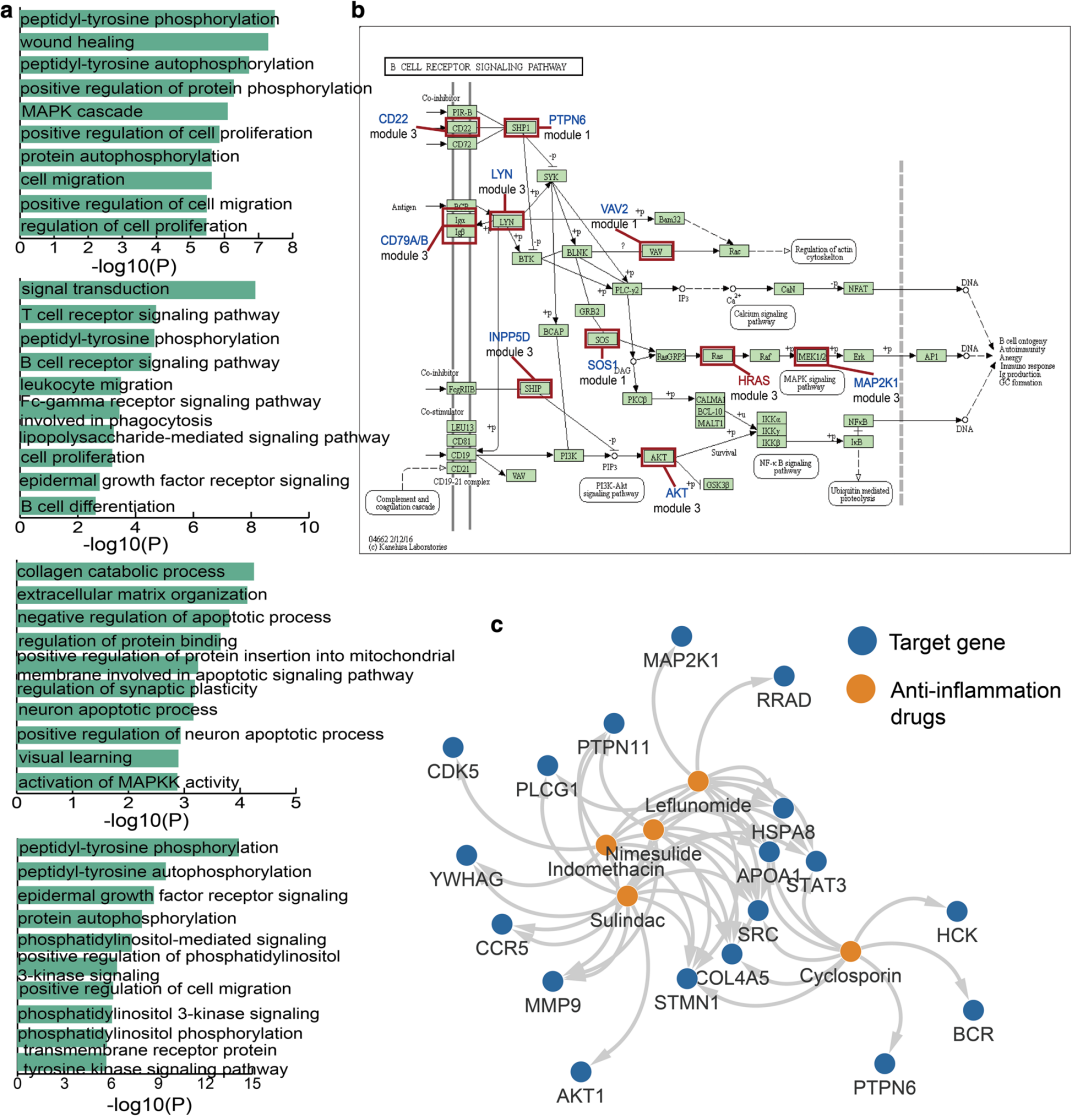
The functional and drug targets analyses for the genes in modules. **a** GO terms enriched for genes in first four modules, respectively, ranked by −log_10_(P) are presented as bar plots. **b** The KEGG pathway B cell receptor signaling pathway and the genes in modules are shown. **c** The network of anti-inflammation and target genes

We perform GO enrichment analysis based on all the genes in the first four modules for MGN. We find these genes in diverse modules are enrichment in some different kinds of GO terms (Fig. 5a). The first and fourth modules are enrichment in some phosphorylation-associated GO terms such as positive regulation of protein phosphorylation, peptidyl-tyrosine phosphorylation and so on. Previous study reports that glomerular cluster in is associated with the phosphorylation of PKC-α/β regulation and good outcome of membranous glomerulonephritis in human [25]. It indicates that the phosphorylation of vascular permeability factor/VEGF-A is essential for the proper maintenance of glomerular filtration barrier and the glomerular endothelial fenestrae follow recent data from podocyte-specific knockout mice as well as studies using neutralizing antibodies [26–29]. Besides, the genes in second module are major enrichment in some GO terms related with immune such as T cell receptor signaling pathway, B cell receptor signaling pathway, B cell differentiation and so on. As we know, some studies suggest that the inhibition of B cell function is associated with beneficial effects on proteinuria in MGN, and human studies clearly demonstrate that the inhibition of B cells with alkylating agents induces remission of the nephrotic syndrome [30, 31]. Experimental results show that T cell subset modulated immunoglobulin production in MGN [32]. The third module are almost enrichment in some GO terms including negative regulation of apoptotic process, positive regulation of neuron apoptotic process and so on. The result of experiment in MGN shows Haeme oxygenase (HO)-1 is considered treat the disease via anti-apoptotic and immunomodulatory effects [33].

Notably, we discover that the genes in these five modules are related with a pathway called B cell receptor signaling pathway (Fig. 5b). In this pathway, we find some immune-related genes including CD22, PTPN6, MAP2K1 and so on and these immune-related genes are key genes for the B cell receptor signaling pathway. In addition, we also find a MGN-related gene HRAS in the third module was play its role in pathway. We also construct an inflammation-related drugs and genes network which is extracted from drug enrichment result (Fig. 5c). The drugs in the network contain Cyclosporin, Indomethacin, Sulindac, Leflunomide and Nimesulide which all could treat inflammation. It also indicates that the essential associations among MGN, immune and inflammation on treatment. In addition, we also get the MGN-related papers for each gene in five modules according to incomplete statistics in pubmed. We found several genes which have been studied in a large number of MGN-related publications due to their important and diverse roles in MGN (Additional file 3: Table S3). The result suggests that the genes in modules can identify genes important in MGN.

## Discussion

Our analyses provide novel insights into the study and treatment of MGN by exploring the functional significance and molecular mechanism of immune-related genes follow interaction network and expression pattern. Although some previous studies identify a part effective biomarkers in MGN, there is no global and system analysis in process [34, 35]. Thus we get all MGN-related genes as far as known and based on these genes perform a global analysis and develop a network-based strategy to identify the MGN-related PPI network and modules.

As we all known, MGN is caused by immune complex formation in the glomerulus. More and more studies suggest that there are strong associations between immune and MGN by diverse research level including cell response, immune therapy, auto-immune and so on [9, 36, 37]. In our analysis of MGN, we focus on the relevance of immune and MGN on gene level. We not only construct immune-related and MGN-related gene interaction network and also integrate expression data of MGN to explore the associations. Our results show that the immune-related genes are closely interacted with MGN-related genes in the network both on network structure and expression level. The differential expression and functional analysis show some immune-related genes could serve as a functional biomarker for MGN.

In this study, we further explore the clinical application of genes in modules for drug development. We find that some genes related with immune and MGN are influenced by drugs for the treatment of inflammation. The immune-related genes may act as functional biomarkers, providing the potential mechanism of action for drugs. Previous study had reported some traditional Chinese medicine such as Skimmin could slow down the progression of MGN by anti-inflammatory effects and inhibiting immune complex deposition [38]. Thus we could study the mechanism of existing anti-inflammatory drugs to develop the novel drugs of treatment MGN. In addition, our method identified novel candidates associated with disease development, which require further research and experimental validation.

Overall, we integrate interaction network and expression data to reveal novel functional insights for immune-related genes in MGN. We discover immune-related genes and MGN-related genes have strong associations not only on network structure but also on expression level. We also identify some immune-related modules in MGN and also show the special expression pattern of these modules. The functional and drug target analysis indicates the mechanism of immune-related genes in MGN.

## Conclusions

In summary, an immune or MGN-directed neighbor network (IOMDN network) and an MGN-related genes-directed network (MGND network) are constructed. By integrating interaction network and expression data, our results show the crucial role of immune-related genes in MGN. Some differential expressed immune-related genes are co-expressed with special differential expressed MGN-related genes, and show some immune-related genes could serve as biomarkers of MGN. The functional analyses further demonstrate the function of immune-related genes in MGN. The network of anti-inflammation drug and target genes provide the novel insights of MGN studies by considering the associations among inflammation, immune and MGN.

## Additional files

**Additional file 1: Table S1.** The gene types of all the genes in IOMGN network.

**Additional file 2: Table S2.** The differential expression details of all genes in five modules.

**Additional file 3: Table S3.** Verified MGN-associated genes in pubmed.

## Abbreviations

MGN: membranous glomerulonephritis
GEO: gene expression omnibus
GO: gene ontology
KEGG: Kyoto Encyclopedia of Genes and Genomes
IOMDN network: immune or MGN-directed neighbor network
MGND network: MGN-related genes-directed network
PPI: protein–protein interaction
PCC: Pearson correlation coefficients
